# Emerging role of a systems biology approach to elucidate factors of reduced penetrance: transcriptional changes in *THAP1*-linked dystonia as an example

**DOI:** 10.1515/medgen-2022-2126

**Published:** 2022-08-12

**Authors:** Sokhna Haissatou Diaw, Fabian Ott, Alexander Münchau, Katja Lohmann, Hauke Busch

**Affiliations:** Institute of Neurogenetics, University of Lübeck, Ratzeburger Allee 160, 23562 Lübeck, Germany; Institute of Experimental Dermatology and Institute of Cardiogenetics, University of Lübeck, 23562 Lübeck, Germany; Institute of Systems Motor Science, University of Lübeck, 23562 Lübeck, Germany

**Keywords:** reduced penetrance, dystonia, DYT-THAP1, iPSC, whole transcriptome

## Abstract

Pathogenic variants in *THAP1* can cause dystonia with a penetrance of about 50 %. The underlying mechanisms are unknown and can be considered as means of endogenous disease protection. Since *THAP1* encodes a transcription factor, drivers of this variability putatively act at the transcriptome level. Several transcriptome studies tried to elucidate THAP1 function in diverse cellular and mouse models, including mutation carrier-derived cells and iPSC-derived neurons, unveiling various differentially expressed genes and affected pathways. These include nervous system development, dopamine signalling, myelination, or cell-cell adhesion. A network diffusion analysis revealed mRNA splicing, mitochondria, DNA repair, and metabolism as significant pathways that may represent potential targets for therapeutic interventions.

## Introduction

**Figure 1:**
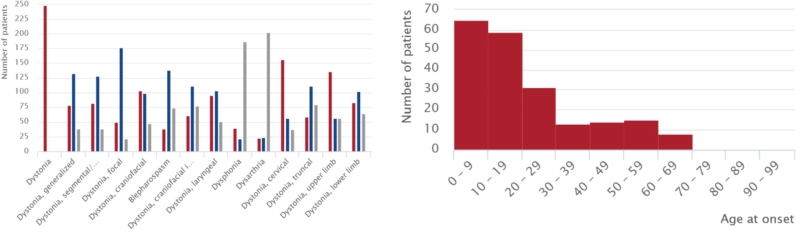
Phenotypic spectrum of reported carriers of *THAP1* mutations. In the left panel, the presence (red) or absence (blue) of the indicated signs and symptoms is shown for 249 patients with *THAP1* mutations. In the right panel, the age variability at onset is illustrated (modified from www.mdsgene.org [4]).

Dystonia is clinically characterized by sustained or intermittent muscle contractions causing abnormal, often repetitive movements, postures, or both [1]. Dystonic movements are typically patterned, twisting, and may be tremulous. Dystonia is a rare disease with a prevalence of about 16 per 100,000 people [2] and may thus affect some 16,000 people in Germany. Within the national research consortium DysTract (http://dystract.cio-marburg.de/de/), information on ∼2,500 of these patients has been collected in a database and DNA biobank for research towards improved understanding of the genetic basis and to develop individualized, i. e., pathophysiology-based, treatment. Within the past 20 years, several genetic forms have been identified for isolated (dystonia as the only disease manifestation) and combined (dystonia in combination with another movement disorder) dystonia [1]. The former includes the *THAP1* (THAP domain-containing apoptosis-associated protein 1) gene [3]. *THAP1-*linked dystonia, previously referred to as DYT6 dystonia, is characterized by early-onset dystonia with prominent craniocervical and upper limb muscle involvement (Figure 1) [4], [5]. Speech impairment due to laryngeal dystonia is common and very characteristic. However, the THAP1-linked phenotype is highly variable, ranging from unaffected carriers to severe generalized dystonia, even within a single family. The disease is inherited in an autosomal dominant fashion with a penetrance of about 50 % [4], [6], [7]. The *THAP1* gene encodes a ubiquitously expressed transcription factor consisting of 213 amino acids and is putatively regulating the expression of various target genes [8], including *TOR1A* [9], the gene mutated in another form of dystonia, and *THAP1* itself [10]. Its DNA-binding properties are associated with the N-terminal THAP domain (amino acids 1–81), including a zinc-finger structure. Towards the C-terminus, THAP1 contains a proline-rich region (amino acids 96–108) and a coiled-coil domain (amino acids 139–190) with a nuclear localization signal (NLS, amino acids 147–162) [11]. To date, ∼100 missense, nonsense, and frameshift mutations in *THAP1* have been described in dystonia patients of different ethnicities [4]. It is believed that the mutations act in a loss-of-function mechanism [12], [13].

Reduced penetrance may reflect endogenous disease protection. Therefore, understanding the underlying factors and processes might open novel therapeutic avenues. However, factors contributing to incomplete penetrance and variable expressivity as well as the disease mechanism of THAP1 dystonia are largely unknown. Despite identical variants in the *THAP1* gene, disease manifestation varies considerably between individuals. Since *THAP1* encodes a transcription factor, it is tempting to speculate that the drivers of this variability are acting at the transcriptomic level accompanied by alterations at the genomic, epigenetic, proteomic, metabolomic, and/or environmental levels. It can be hypothesized that identifying protective variants will enable expansion of genetic testing and thus a more sophisticated prediction of the disease course in *THAP1* mutation carriers. Further, the elucidation of altered (transcriptional) networks that contribute to disease protection can unravel potential therapeutic targets by manipulating these networks in the desired direction using, for instance, small molecules.

## Transcriptional studies

Initial attempts to understand the role of *THAP1* in transcriptional regulation were carried out before establishing the disease link to dystonia. At that time, researchers investigated transcriptional changes in endothelial cells [8]. They linked *THAP1* function to cell cycle control: Retroviral-mediated gene transfer of *THAP1* in primary human endothelial cells inhibited proliferation and G1/S cell-cycle progression. *THAP1* overexpression downregulated > 50 genes encoding proteins associated with cell-cycle/cell proliferation [8]. Further, a few other differentially expressed genes (DEGs) were linked to diverse biological functions. siRNA-mediated *THAP1* knock-down resulted in inhibition of S-phase DNA synthesis. Of note, this early study had already indicated downregulation of *THAP1* when over-expressed [8], the mechanism of which, i. e., autoregulation, was resolved later [10].

To unravel the THAP1-mediated disease mechanism in dystonia, the search for neuronal targets and DEGs is ongoing, and different cellular and mouse models have been established. In mutant or overexpression *THAP1* models, it is expected that target expression will be altered. Meanwhile, several dysregulated genes have been found using these models. Table 1 summarizes relevant candidate DEGs from unbiased transcriptome studies [8], [14], [15], [16], [17], [18], [19], [20], [21], [22].

**Table 1:** Overview of THAP1 transcriptional expression and regulation in various research models.

Gene	Functions	Direction	Model	self-reported significance (p-value)	Tissue	References
*AARS2*	The mitochondrial enzyme that plays critical roles in mRNA translation by charging tRNAs with their cognate amino acids	UP	THAP1 Nestin conditional knockout mice	***	cerebellar and striatal tissue	14
*AGPAT1*	converts lysophosphatidic acid (LPA) into phosphatidic acid (PA); LPA and PA are two phospholipids involved in signal transduction and lipid biosynthesis in cell	UP	cortical neuronal precursors derived from human iPSC	***	in vitro	17
*ANLN*	actin-binding protein that plays a role in cell growth, cell migration, and cytokinesis	DOWN	THAP1 Nestin conditional knockout mice	**	cerebellar and striatal tissue	14
*ATF4*	transcription factor essential for stress-induced autophagy gene expression; key effector of the eIF2α pathway	DOWN	THAP1^+/-(ΔExon2)^ knockout mice; Flp-In T-REx 293 cells	***	striatum; in vitro	15, 18
*ATP5F1E*	subunit of mitochondrial ATP synthase	UP	cortical neuronal precursors derived from human iPSC	***	in vitro	17
*BDKRB2*	receptor for bradykinin; the 9 aa bradykinin peptide induces responses like vasodilation, pain fiber stimulation, edema, and smooth muscle spasm	DOWN	cortical neuronal precursors derived from human iPSC	***	in vitro	17
*CLN3*	protein involved in lysosomal function; mutations cause a neurodegenerative disease known as Batten disease	DOWN	THAP1 Nestin conditional knockout mice	***	cerebellar and striatal tissue	14, 16
*CNP*	myelin protein; *THAP1* regulates myelination	DOWN	THAP1 Nestin conditional knockout mice	**	striatum	16
*CRADD*	protein containing a death domain (DD) motif; it recruits caspase 2/ICH1 to the cell death signal transduction complex and promotes apoptosis; mutation in this gene is associated with cognitive disability	DOWN	THAP1 Nestin conditional knockout mice	***	cerebellar and striatal tissue	14
*CUEDC2*	down-regulates ESR1 (estrogen receptor) protein levels, and it is involved in the 17 beta-estradiol-induced ESR1 degradation; it controls PGR protein (progesterone receptor) levels	DOWN	THAP1 Nestin conditional knockout mice	***	cerebellar and striatal tissue	14, 16
*CYB5D2*	promotes neuronal differentiation through inhibiting cell proliferation	UP	THAP1 Nestin conditional knockout mice	***	cerebellar and striatal tissue	14
*DNLZ*	zinc finger protein that may function as a co-chaperone towards HSPA9/mortalin, which is prone to self-aggregation	UP	THAP1 Nestin conditional knockout mice	***	cerebellar and striatal tissue	14
*DPAGT1*	enzyme that is an integral membrane protein of the endoplasmic reticulum that catalyzes the first phase for glycoprotein biosynthesis	DOWN	THAP1 Nestin conditional knockout mice	***	striatum	16
*DPH6*	catalyzes the last step of diphthamide biosynthesis using ammonium and ATP	DOWN	THAP1 Nestin conditional knockout mice	***	cerebellar and striatal tissue	14
*DRD2*	plays a critical role in the indirect pathway of the basal ganglia; *DRD2* codes for one of two DA receptors expressed by striatal medium spiny neurons	DOWN	THAP1 Nestin conditional knockout mice	***	cerebellar and striatal tissue	14
*DRD4*	receptor that may play a role in mediating reduced penetrance in *THAP1* mutation carriers	UP	cortical neuronal precursors derived from human iPSC	***	in vitro	17
*ECH1*	member of the hydratase/isomerase superfamily; it is involved in the pathway fatty acid beta-oxidation	DOWN	THAP1 Nestin conditional knockout mice	***	cerebellar and striatal tissue	14, 16
*EHD3*	ATP- and membrane-binding protein that controls membrane reorganization/tubulation upon ATP hydrolysis; plays a role in endocytic transport	DOWN	THAP1 Nestin conditional knockout mice	***	cerebellar and striatal tissue	14
*EIF3K*	plays an essential role in translation by binding directly to the 40S ribosomal subunit and promoting the formation of the 40S preinitiation complex	DOWN	THAP1^+/-(ΔExon2)^ knockout mice	**	cerebellar tissue	15
*EIF4G3*	The protein part of the eIF4F protein complex, which is involved in mRNA cap recognition and transport of mRNAs to the ribosome; downregulation leads to a general decrease in cell proliferation and protein translation	UP	cortical neuronal precursors derived from human iPSC	***	in vitro	17
*ElF2α*	elF2α signalling, involved in translational regulation, is a generalizable mechanism for dystonia; *THAP1* mutations in neonatal mouse striatum and cerebellum identified eIF2α signalling as one of the top dysregulated pathways	DOWN	THAP1^+/-(ΔExon2)^ knockout mice	**	in vitro; cerebellar tissue	15
*FAM117A*	codes for a protein of unknown function	UP	THAP1 Nestin conditional knockout mice	*	cerebellar and striatal tissue	14
*FAM122B*	codes for a protein of unknown function, seems to be highly expressed in brain	UP	THAP1 Nestin conditional knockout mice	*	cerebellar and striatal tissue	14
*HLA-A*	expressed in nearly all cells, this gene plays a central role in the immune system by presenting peptides derived from the endoplasmic reticulum lumen	DOWN	cortical neuronal precursors derived from human iPSC	***	in vitro	17
*LPAR1*	key mediator of diverse biologic functions, such as proliferation, cell differentiation, inhibition of neuroblastoma, chemotaxis, and smooth muscle contraction	DOWN	oligodendrocytes of THAP1 Nestin conditional knockout; THAP1^C54Y/+^ mice	*	in vitro; striatum	16, 21
*LYRM1*	member of the mitochondrial leucine/tyrosine/arginine motif family of proteins; overexpression in adipocytes causes abnormal mitochondrial morphology and mitochondrial dysfunction	UP	THAP1 Nestin conditional knockout mice	**	cerebellar and striatal tissue	14
*MAG*	myelin protein; *THAP1* regulates myelination	DOWN	oligodendrocytes of THAP1 Nestin conditional knockout; THAP1^C54Y/+^ mice	**	in vitro; striatum	16
*MOBP*	myelin component; *THAP1* appears to be essential for the expression of *MOBP*	DOWN	oligodendrocytes of THAP1 Nestin conditional knockout	***	in vitro	16
*MOG*	myelin protein	DOWN	oligodendrocytes of THAP1 Nestin conditional knockout	***	in vitro	16
*PLLP*	member of the group of myelin proteins; this membrane protein is linked to human hereditary demyelinating neuropathies	UP	oligodendrocytes of THAP1 Nestin conditional knockout	**	in vitro	16
*PLP1*	myelin protein	DOWN	oligodendrocytes of THAP1 Nestin conditional knockout	**	in vitro	16
*PPP2R3C*	regulatory subunit of the serine/threonine phosphatase, protein phosphatase 2; may regulate the expression of the P-glycoprotein ATP-binding cassette transporter through its phosphatase activity	UP	THAP1 Nestin conditional knockout mice	**	cerebellar and striatal tissue	14
*RACGAP1*	GTPase-activating protein (GAP), a component of the centralspindlin complex; plays a regulatory role in cell growth, differentiation, and cytokinesis.	UP	THAP1 Nestin conditional knockout mice	***	cerebellar and striatal tissue	14
*RRM1*	direct transcriptional target of THAP1 that modulates it	DOWN	primary striatal neurons; endothelial cells; HUVECs	*	in vitro	8, 22
*RRM1*		UP	THAP1^C54Y/+^ mice	**	striatum	21
*SHLD1*	DNA end-protecting activity; inactivation of *THAP1* abolishes *SHLD1* expression, and impairment of THAP1-dependent Shld1 expression could result in the accumulation of neuronal DSBs	DOWN	THAP1 Nestin conditional knockout mice	***	cerebellar and striatal tissue	14, 20
*SIGLEC1*	type I transmembrane protein is expressed only by a subpopulation of macrophages; it is involved in mediating cell to cell interactions	DOWN	cortical neuronal precursors derived from human iPSC	***	in vitro	17
*SIX2*	plays an important role in brain development; knockdown of this gene leads to decreased cell viability and increased apoptosis of damaged dopaminergic neurons	DOWN	cortical neuronal precursors derived from human iPSC	***	in vitro	17
*SLC6A13*	permits efflux of GABA and taurine from the brain to the circulating blood through the blood-brain barrier; one of its key functions is to influence the activity of neurotransmitter GABA in the brain	UP	THAP1^C54Y/+^ mice	*	cerebellar tissue	21
*SLX4IP*	involved in DNA repair and maintenance	UP	THAP1 Nestin conditional knockout mice	**	cerebellar and striatal tissue	14
*SNRNP35*	A homolog of U1-snRNP binding protein that participates in processes like RNA splicing and mRNA processing	UP	THAP1 Nestin conditional knockout mice	***	cerebellar and striatal tissue	14
*SOD2*	member of the iron/manganese superoxide dismutase family; it expresses a mitochondrial protein	DOWN	HEK-293 and SK-N-AS cells	*	in vitro	19
*STXBP1*	syntaxin-binding protein that plays a role in the release of neurotransmitters via regulation of syntaxin, a transmembrane attachment protein receptor	DOWN	HEK-293 and SK-N-AS cells	**	in vitro	19
*THAP1*	cause of DYT-THAP1	DOWN	cortical neuronal precursors derived from human iPSC; human fibroblasts; THAP1 Nestin conditional knockout mice; endothelial cells	**, ***	cerebellar and striatal tissue	8, 14, 17
*TOMM40*	localized in the outer membrane of the mitochondria, it is the channel-forming subunit of the translocase of the mitochondrial outer membrane (TOM) complex that is essential for the import of protein precursors into mitochondria.	UP	THAP1 Nestin conditional knockout mice	**	cerebellar tissue	14
*TOMM40*		DOWN	THAP1 Nestin conditional knockout mice	***	striatum	14
*TOR1A*	wild-type *THAP1* represses the expression of TOR1A; contrarily mutant *THAP1* results in decreased repression of *TOR1A*	UP	SH-SY5Y cells; human fibroblasts; HEK-293T, HUVECs and T98G cells	N/A	in vitro	7, 9, 21
*TSPAN2*	member of the transmembrane 4 superfamily; it mediates signal transduction events that play a key role in the regulation of cell activation, development, and motility	DOWN	oligodendrocytes of THAP1 Nestin conditional knockout	***	in vitro	16
*UGT8A*	abundant sphingolipid of the myelin membrane of the central nervous system and peripheral nervous system	DOWN	oligodendrocytes of THAP1 Nestin conditional knockout	***	in vitro	16
*YY1*	transcription factor ubiquitously distributed, belonging to the GLI-Kruppel class of zinc finger proteins; it is involved in repressing and activating many promoters	UP	cortical neuronal precursors derived from human iPSC; oligodendrocytes of THAP1 Nestin conditional knockout	***	in vitro	16, 17
*ZFP882*	zinc finger protein highly expressed in the brain of mice, and transcriptional regulator	DOWN	THAP1 Nestin conditional knockout mice	**	cerebellar and striatal tissue	14

Significance: * = p value < 0.05, ** = p-value < 0.01 and *** = p-value < 0.001.

By this long list of DEGs and the diverse pathways affected by dysfunctional THAP1, the role of THAP1 seems to be manifold: Transgenic mice expressing heterozygous loss-of-function THAP1 showed alterations in the expression of genes involved in nervous system development [14]. *THAP1* is also necessary for the timing of myelination initiation in oligodendrocytes [16]. Further, dysregulation of genes involved in the eIF2α (Eukaryotic Initiation Factor 2 alpha) signalling pathway, mitochondrial dysfunction, and neuron projection development have been observed in the brains of THAP1^+/-(ΔExon2)^ knockout mice [15]. An essential role for THAP1 in cell survival and proliferation has been demonstrated in murine embryonic stem cells [23]. Moreover, it was found that wild-type *THAP1* regulates genes involved in cell growth and proliferation in neuronal cells, while mutant *THAP1* leads to the dysregulation of genes related to synaptic function, a process that has been reported as a pathogenic mechanism of other subtypes of dystonia [19]. In patient-derived cortical neurons, dopamine signalling seemed to be altered and involved in disease expression [17].

## DNA-binding of the transcription factor THAP1

It is thought that transcriptional regulation via THAP1 is mediated by binding of THAP1 to promoter regions within the nuclear DNA. There is a significant co-binding with other transcription factors, such as HCFC1 [24] or YY1 [16], as also underlined by the ENCODE CHIP-Seq studies. *YY1* encodes another transcription factor with a recognized and established role in myelination; pathogenic variants in *YY1* cause a severe neurodevelopmental disorder [25] as well as early-onset dystonia [26], [27]. It has been shown that THAP1 modulates the DNA occupancy of YY1 in non-conditional knockout mice and that loss of *THAP1* impairs myelination in the central nervous system via a cell-autonomous role in decreased DNA occupancy and oligodendrocyte lineage [16]. Of note, *YY1* has been found to be upregulated in cortical neurons of manifesting when compared to non-manifesting mutation carriers or healthy controls [17]. The low percentage (around 10 %) of overlap between RNA-Seq and ChIP-Seq datasets may highlight the role of important *THAP1* co-factors like YY1 and HCFC1 and indicate a different role of *THAP1* in regulating gene expression other than direct binding at DNA, as in other zinc-finger factors [23].

Further, *THAP1* ChIP-Seq analysis in neuronal cells overexpressing *THAP1* revealed that THAP1 is able to bind and activate promoter regions of different *SOD2* isoforms in SK-N-AS human neuroblastoma cells [19]. Knockout of *SOD2* in mice seems to impair mitochondrial enzyme activity leading to elevated reactive oxygen species (ROS) content in synaptosomes, altering synaptic function [28]. This discovery is a possible means of how *THAP1* mutations cause an expressional change of genes related to synaptic function.

## Pathway analyses in the pathogenesis of THAP1

To put the different DEGs from the various *THAP1* studies into a broader picture, gene enrichment analysis has been performed using Gene Ontology (GO) terms or KEGG pathways. Affected pathways are diverse and include peripheral nervous system development, cytoskeleton, neuron projection development, dopamine signalling, myelination, axonal guidance, long-term synaptic depression, cell-cell adhesion, gliogenesis, and muscle movement and spasm [14], [15], [17], [29]. In fact, higher brain regions such as those underlying sensorimotor function may be dysfunctional, acting jointly with abnormalities attributable to the noradrenergic system originating in the locus coeruleus of the brainstem.

The link to dopamine signalling seems particularly interesting and strong: Links between dystonia and dopamine are numerous [30], and although isolated dystonia, especially DYT-THAP1, is usually not responsive to dopaminergic treatment, recent data suggest that *DRD4* expression levels may play a role in mediating penetrance in *THAP1* mutation carriers [17]. *DRD4* is a member of the dopamine D2-like receptor family, characterized by its ability to inhibit adenylyl cyclase. *DRD2* encodes a dopamine receptor expressed by striatal medium spiny neurons and plays a critical role in the indirect pathway of the basal ganglia. Recently, DRD2 was demonstrated to have a fundamental role in motor control and balance in knockout mice’s medium spiny neurons and cholinergic interneurons [31]. Further, *DRD2* may be one of the indirect targets involved in the pathogenic pathways disrupted by *THAP1* transcriptional deficit [14]. Other studies also provided evidence for changes in the dopamine signalling pathway as one of the top hits among several neurotransmitter-linked pathways upregulated in dystonia [14], [17]. A subnetwork of differentially regulated genes connected to cell cycle regulation and neurogenesis has been identified and may provide a molecular explanation for the disrupted dopaminergic neurotransmission and neuronal biogenesis in the pathogenesis of dystonia [31].

Further, a significant downregulation of genes related to apoptosis, including *CRADD* (*CASP2 And RIPK1 Domain Containing Adaptor with Death Domain*), *SIX2* (*Homeobox protein SIX2*), and a significant dysregulation of genes implicated in autophagy and mitochondrial homeostasis including *ATF4* (*Activating Transcription Factor 4*), *LYRM1* (*LYR Motif Containing 1*) and *SOD2* (*Superoxide dismutase 2*) were observed in cortical neuronal precursors derived from human iPSC as well as in mice [14], [15], [17].

Most studies on differential gene expression in neuronal cells also revealed a transcriptional signature that point to *THAP1* as a regulator of inflammatory responses by regulation of Interleukin-5 and Interleukin-6 production [14], [16], [17], [21]. Further, GO analyses of biological processes of upregulated genes in murine embryonic stem cells revealed terms related to embryonic pattern specification, chromosome organization, meiosis, and negative regulation of cell differentiation [23]. Analysis of down-regulated genes in transgenic mice and murine embryonic stem cells proved enrichment for processes involved in neuronal development like axonogenesis, differentiation of neurons, and cell projection assembly and organization [14], [23].

**Figure 2:**
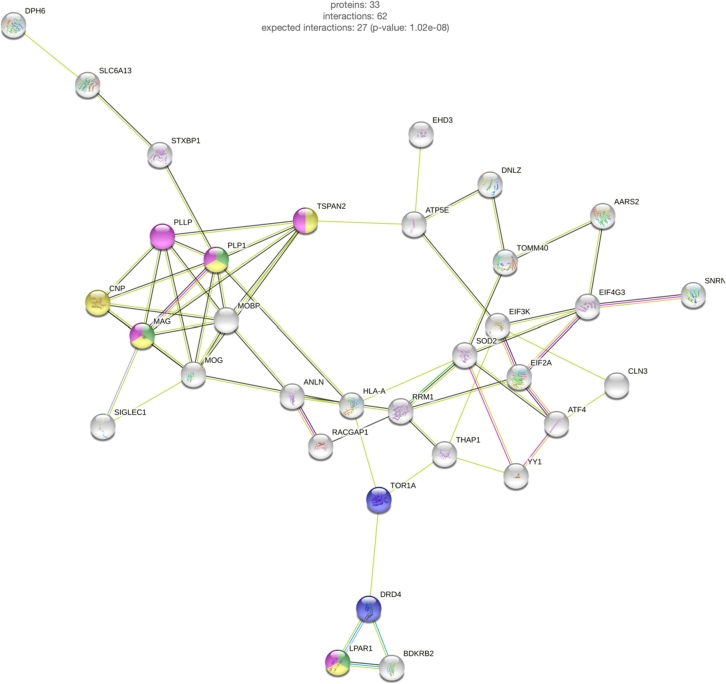
Protein Interaction Network related to THAP1. Interactions of the THAP1-mediated DEGs were obtained via StringDB. The colors of the nodes symbolize the affiliation to specific GO:terms, colors of the edges symbolize the type of protein interaction. The most frequent interaction among these genes is related to data mining in current studies (light yellow border). Only individual clusters are linked via co-expression (black edge) or experimental evidence.

Another pathway that repeatedly came up as being regulated by *THAP1* was the eIF2α pathway in mouse and human models. Interestingly, the elF2α pathway seems to be involved in the pathogenesis of different (monogenic) forms of dystonia, such as DYT-TOR1A or DYT-PRKRA, and probably also *SGCE*-linked myoclonus dystonia [17], [32]. The most recent evidence for the role of the elF2α pathway stems from the discovery of pathogenic variants in a member of the eIF2α kinases family, *EIF2AK2* (*Eukaryotic translation initiation factor 2 alpha kinase 2*), in early-onset generalized dystonia [32]. In addition to being a key component of Endoplasmic Reticulum (ER) stress responses and synaptic plasticity, the eIF2α signalling also regulates important physiological events under homeostatic conditions like the accumulation of misfolded proteins [15]. Thus, the eIF2α dysregulation may represent a point of convergence between different forms of dystonia through its influence on critical homeostatic neurodevelopmental events. Therefore, it is conceivable that eIF2α signalling is involved in the expressivity of *THAP1* mutations [15]. Interestingly dysregulation of the eIF2α pathway has been observed in cortical neurons and fibroblasts of dystonia patients as well [17].

Several of the affected pathways have recurrently been implicated in response to alterations of THAP1. To test for the functional relatedness of all the studies mentioned above and to find regulatory interactions of all the identified THAP1-associated genes and proteins in an unbiased way, we mapped the genes from Table 1 on the protein interaction network from StringDB [33] (Version 11, confidence cutoff score 0.7). This database includes both known and predicted protein-protein interactions of various organisms that are inferred from direct physical and indirect functional associations from high- and low-throughput experiments, as well as computational prediction.

**Figure 3:**
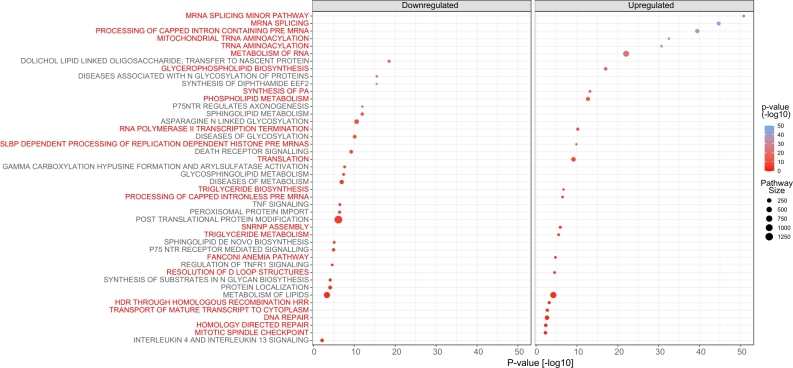
Hypergeometric test on Reactome pathways of proteins affected by differential gene expression in THAP1 knockdown models. The dot plot includes pathways having at least three affected proteins at a significance cutoff of p < 0.05. Red and black font colors refer to up- and down-regulated pathways, respectively. The dot size corresponds to the pathway set size.

In total, 45 genes were assigned to the human protein-protein interaction network, with 33 proteins sharing 62 interactions (Figure 2). Interestingly, we would expect only 27 interactions from a network of 45 randomly picked proteins. Thus, we have significantly higher connectivity of THAP1-associated proteins ($\text{p-value}=1.02\times 10^{-8}$), which indicates their functional relatedness that should be investigated further.

To further investigate the function of the differentially regulated transcripts and proteins in combination with their neighbouring interacting proteins, we performed a network diffusion on the protein-protein interaction network from StringDB using the R library diffuStats [34]. Network diffusion assumes that the effect of differential gene regulation also spreads to the neighbours on a protein-protein interaction network. The effect size is calculated by “diffusing” the magnitude of differential regulation across the protein interaction network until a steady-state is reached. Considering all affected nodes above a certain diffusion score then provides a broader view of the molecular function affected by the initial gene set. Here we considered both the significance and direction of differential regulation of the 45 mapped genes by a $\log_{10}$ transform of the reported p-values and the sign according to up- or downregulation. Network diffusion was done on a regularised Laplacian kernel, and the 1 %, or respectively 169, proteins having the most positive or negative diffusion scores were investigated separately for pathway enrichment by a hypergeometric test [35]. Among the proteins affected by upregulated transcripts, we found mRNA splicing, mitochondria, DNA repair, and metabolism as the most significant pathways, while glycosylation, axonogenesis, sphingolipid, death receptor, and TNF signalling seem to be downregulated (Figure 3). While transcriptome analyses repeatedly revealed differential gene regulation correlated with cell cycle regulation, neurogenesis, inflammatory responses, and cell death in different cell and mice models of THAP1-DYT, these overarching analyses expand the pathways that may play a role in the pathophysiology of (THAP1) dystonia and warrant further studies and validation.

## Conclusions and outlook

THAP1 activities are likely due to the regulation of gene expression via its role as a transcription factor. However, THAP1 downstream targets in neurons, the mechanism via which THAP1 mutations cause disease, and the disease mechanism underlying isolated dystonia, in general, are all largely unknown. There are no reported *THAP1* genotype-phenotype-predictors. A systems biology approach that not only focuses on a single isolated component of the regulatory mechanisms may help to shed further light on dysregulated pathways underlying THAP1 dystonia. These insights will probably allow predictions as to the likely clinical course in *THAP1* mutation carriers and are also expected to foster the development of disease-modifying treatments with the ultimate aim of individualized therapeutic strategies. Thus, certain systems biology patterns may be associated with an overall favorable outcome justifying a “wait and see” strategy, others may indicate the preference for a more severe clinical course necessitating more rigorous management. DYT-THAP1 is likely caused by an interplay of molecular aetiologies that are poorly understood, thereby limiting the efforts of designing functional assays that could be utilized to screen for novel therapeutics. In this context, identifying pathways impacted by THAP1 mutations as factors influencing penetrance and expressivity should be prioritized. These pathways may represent the best potential targets for therapeutic intervention, which could eventually offer widespread benefit to a broad and diverse population of dystonia patients.
